# Putative Markers of Repression in Patients Suffering From Mental Disorders

**DOI:** 10.3389/fpsyg.2018.02109

**Published:** 2018-11-06

**Authors:** Aram Kehyayan, Nathalie Matura, Kerstin Klein, Anna-Christine Schmidt, Stephan Herpertz, Nikolai Axmacher, Henrik Kessler

**Affiliations:** ^1^Department of Psychosomatic Medicine and Psychotherapy, LWL University Hospital, Ruhr-University Bochum, Bochum, Germany; ^2^Department of Neuropsychology, Faculty of Psychology, Institute of Cognitive Neuroscience, Ruhr-University Bochum, Bochum, Germany

**Keywords:** free association, repression, psychodynamic conflict, skin conductance response, patients, operationalized psychodynamic diagnosis, psychoanalysis, neuropsychoanalysis

## Abstract

**Background:** The concept of psychodynamic conflict is essential to psychodynamic theory and therapy. In classical psychodynamic therapy, unconscious conflict themes need to be identified by the therapist and brought to the patient’s awareness, in order to work through and ultimately solve them. According to theory, touching upon conflict-related topics leads to arousal, followed by activation of defense mechanisms such as repression. Starting with C.G. Jung’s association studies more than 100 years ago, various proposals have been made to investigate psychodynamic conflicts based on free association and psychophysiological measures. This study presents an attempt to identify and differentiate between psychodynamic conflict themes in patients, using an adopted version of Jung’s paradigm that had in previous studies been applied to healthy subjects.

**Method:** Seventeen patients suffering from depression and other mental disorders associated freely to different cue sentences. Prior to the experimental procedure, patients’ individual psychodynamic conflict types were assessed through clinical interviews. Sentences were either neutral, negative (but not conflict-related), or related to specific types of psychodynamic conflicts. Memory for the first three associations was later tested in an unexpected recall task. Skin conductance response (SCR) was recorded and analyzed together with reaction times (RTs) and self-ratings of emotional valence, arousal, and agreement with cue sentences.

**Results:** Patients showed reduced memory performance for associations to conflict-related sentences in general, compared with negative and neutral sentences. Agreement with conflict-related sentences was lower compared to neutral but not negative sentences. Memory was negatively correlated with RTs and SCR. RTs were longer for conflict types that had been rated as relevant in clinical interviews prior to the association task, compared to the other, non-relevant conflict types.

**Conclusion:** Our study shows that some putative markers of repression of psychodynamic conflicts previously established in healthy participants also occur in patients. Moreover, it provides evidence that general conflict effects differ from specific effects of personally relevant conflicts.

## Introduction

Psychodynamic conflicts constitute a central part of psychoanalytic and psychodynamic theory (Brenner, 1982; Mentzos, 1992; Person et al., 2005; OPD Task Force, 2008). According to Freud and other scholars, unresolvable conflicts between incompatible needs or desires can cause unbearable anxiety (“Signalangst”), leading to activation of defense mechanisms in order to ward off conscious awareness of these conflicts. This, however, comes with a cost: The conflict is not resolved, but continues to exist unconsciously, resulting in conflict tension. Mental disorders or symptoms, in this view, serve to discharge and therefore alleviate conflict tension. This is what Freud called *neurosis*. Importantly, a specific conflict type is not inherently connected with a specific symptomatology, and reversely a specific set of symptoms may be due to different types of conflict or could have a primarily non-conflictual (e.g., structural) etiology. For example, a major depression could be caused by narcissistic insults (possibly activating a pre-existing conflict of self-value), or alternatively by the feeling of receiving too little attention or care from friends, colleagues, or family (reflecting a conflict of “desire for care vs. autarchy”). On the other hand, a psychodynamic conflict centered around, e.g., themes of desire for care vs. autarchy can lead to a number of different symptoms, like anxiety or somatoform symptoms.

Psychodynamic therapies aim at alleviating these symptoms through identification and, eventually, resolution of underlying pathogenic psychodynamic conflicts. One of the main techniques used especially in psychoanalytic treatments is *free association* (Freud, 1913/1958). Patients are encouraged to say whatever comes to their mind without holding back any content, no matter how meaningless or embarrassing it may seem. According to Freud, free association lowers censorship, i.e., the internal force dedicated to keeping unconscious contents from entering into consciousness and causing anxiety. Because this lowering of censorship poses a threat to psychic stability, opposing *resistance* phenomena can frequently be observed in the course of treatments: emotional reactions, arousal, or delays in the flow of associations can indicate the appearance of contents which may be linked to unconscious (repressed) conflicts.

Since the beginnings of psychoanalysis and throughout the twentieth century, psychoanalytic constructs and explanations have been subject to criticism from different directions (e.g., Popper, 1962; Hobson and McCarley, 1977; Eysenck, 1985; Grünbaum, 1988). One rather fundamental criticism, in essence, argues that psychoanalytic theory is unscientific because important processes (like repression) cannot be directly observed, and because explanations for their underlying causes were considered unfalsifiable (see Axmacher, 2013, for a response to this criticism). On the other hand, there have been numerous attempts to find empirical evidence supporting psychoanalytic claims. Although rather diagnostically than theoretically oriented in nature, C.G. Jung examined the relationship between resistance and repression in patients undergoing psychoanalysis in his association studies (Jung, 1906). Presenting subjects with a list of stimulus words and asking them to name the first word coming to their mind, he discovered that some associations were generated with a delay (longer reaction time, RT) and accompanied by higher Galvanic Skin Responses [also called Skin Conductance Response (SCR) or Electrodermal Activity (EDA)]. Jung interpreted these reactions, together with a failure of his subjects to reproduce these associations, as signs of resistance, encouraging him to explore with his patients thematic fields related to the respective stimuli or associations in order to uncover unconscious psychodynamic conflicts. Several adaptions of Jung’s paradigm showed that associations generated with longer RTs and accompanied by higher SCRs were less likely to be remembered in a surprise memory task, possibly indicating a fast occurring re-repression of conflict-related material (Levinger and Clark, 1961; Köhler and Wilke, 1999).

In several previous studies based on Jung’s experiments (Kehyayan et al., 2013; Schmeing et al., 2013; Kessler et al., 2017), we developed a free association paradigm in which healthy subjects are confronted with conflict-related and non-conflict-related sentences with the task to freely associate in response. After each trial, subjects rate their mood (in terms of valence and arousal) and their level of agreement with the stimulus sentence (“How strongly does the sentence apply to you personally?”). These studies provided evidence for the claim that free association specifically to psychodynamic conflicts elicits behavioral and psychophysiological reactions that could be interpreted as resistance and, in case of subsequent memory failure, as repression. In these studies, associations to conflict-related sentences showed longer reaction times, higher SCRs, and were less likely to be subsequently remembered, compared to non-conflict-related sentences. The content of associations was also analyzed qualitatively by trained psychotherapists, who identified trials in which the stimulus had actually triggered psychodynamic conflicts (which were likely to be non-pathological, as our sample consisted of healthy volunteers). Subjects who showed such signs of psychodynamic conflict in at least one trial reported higher levels of agreement with the contents of conflict-related stimulus sentences in general (i.e., they rated these sentences as applying to them more strongly). These subjects also reported a more negative mood in response to these sentences. Moreover, these participants also showed specific responses to these putatively relevant conflicts (according to clinical ratings) as compared to other conflict sentences, since they were accompanied by higher ratings of agreement and more negative mood.

Identifying relevant psychodynamic conflicts remains an important task for analysts and psychodynamic therapists. The system of Operationalized Psychodynamic Diagnostics (OPD; OPD Task Force, 2008) defines seven thematically distinct conflict types and provides considerations on how to identify these conflicts in a diagnostic interview. This system provides a reliable and widely used approach to assess psychodynamic conflicts, but is relatively time-demanding and not based on quantifiable experimental assessments. Is there another, possibly easier way to identify psychodynamic conflicts and to experimentally validate psychodynamic conflict hypotheses generated in diagnostic interviews or throughout therapy?

The present study strives to answer these questions. It is our first application of the free association paradigm in a patient population. We hypothesized to replicate our findings from previous studies, possibly with stronger effects, as we expected psychodynamic conflicts to be more pronounced in patients than in healthy subjects. In detail, we expected (1) memory performance to be impaired for associations to conflict-related compared to non-conflict-related sentences, (2) longer reaction times as well as more pronounced psychophysiological reactions following presentation of conflict-related compared to non-conflict-related sentences, and (3) correlations between memory performance, RT and measures of psychophysiological arousal, respectively. Also, as specific psychodynamic conflicts were identified through clinical interviews and categorized based on OPD in all patients, we wanted to validate the ability of our paradigm to differentiate between different types of psychodynamic conflicts. Specifically, we expected (4) impaired memory performance for associations to conflict-related sentences targeting patients’ relevant conflict type(s) compared to other conflict types, and (5) longer reaction times and more pronounced psychophysiological reactions (SCR) following presentation of personally relevant vs. non-relevant conflict-related sentences. If successful, the free association paradigm could provide further support for the concepts of psychodynamic conflicts, resistance, and repression, and prove useful as an add-on tool to identify or differentiate between psychodynamic conflicts in patient populations undergoing psychodynamic or psychoanalytic treatments, based on behavioral (memory, RT) and psychophysiological (SCR) reactions.

## Materials and Methods

### Ethics Statement

The study was approved by the Ethics Committee of the Medical Department at the Ruhr-University Bochum, Germany (“Ethik-Kommission der Medizinischen Fakultät der Ruhr-Universität Bochum”; Reg.-Nr. 5083-14). It was in accordance with the latest version of the Declaration of Helsinki, and all subjects provided written informed consent.

### Participants

Participants were recruited both from the outpatient and inpatient units of the Department of Psychosomatic Medicine and Psychotherapy at the LWL University Hospital in Bochum, Germany. Patients had to be between 18 and 65 years of age and to suffer from mental disorders from the domain of psychosomatic medicine and psychotherapy, including depressive disorders, anxiety disorders, somatoform (and somatoform pain) disorders, eating disorders (excluding anorexia nervosa), obsessive-compulsive disorder, and psychological and behavioral factors associated with somatic disorders or diseases (e.g., problems in accepting and dealing with diabetes). Patients suffering from posttraumatic stress disorder (PTSD) were excluded from participation, because PTSD is by definition believed to be primarily caused by traumata rather than psychodynamic conflicts. Also, patients with anorexia nervosa were not included in this study due to known changes in autonomic nervous system functioning (Mazurak et al., 2011), which could limit the possibility to discriminate levels of arousal. All patients were diagnosed in clinical interviews conducted by experienced therapists in the in- and outpatient units of the hospital. In addition to symptom-based diagnoses, therapists assessed the psychodynamic conflicts using Operationalized Psychodynamic Diagnostics (OPD; OPD Task Force, 2008). Patients were only included in the study if therapists indicated the likely existence of at least one out of three types of psychodynamic conflict: desire for care vs. autarchy; conflicts of self-value; or submission vs. control. These specific conflicts were chosen (out of seven conflict types defined in the OPD) because they are seen most frequently in patients, based on our clinical experience. These conflicts are described in greater detail further below. A total of *N* = 17 participants were recruited (8 females), with a mean age of 43.5 years (SD 10.0 years; range: 23–57 years). All but one patient suffered from depressive symptoms, with comorbid diagnoses from the areas mentioned above. Table 1 provides a detailed description of the patients. While behavioral measures (memory performance, reaction times, self-ratings) were obtained from all patients, SCR measures were only obtained from 14 patients due to technical problems with the measuring device.

**Table 1:** Patient overview.

Age	Sex	Mode of recruitment	ICD-10 diagnoses	Estimated conflict	
				CA	SV
44	M	O	F33.0	X	X
57	F	O	F32.1, F54, J45.9		X
45	M	O	F33.1, F45.41, F54, K86.1	X	
50	M	O	F33.1, F45.41, F54, L40.0	X	X
23	M	O	F32.1, F45.30		X
57	M	I	F33.1, F45.1		X
32	F	I	F32.1, F50.9	X	X
50	F	O	F32.1	X	
51	F	I	F33.0, F50.9, F54, E11.90	X	X
31	M	O	F33.2, F63.8		X
48	F	I	F33.1, F60.7	X	
34	M	I	F32.1, F50.9	X	X
39	F	I	F32.1, F40.2, F40.01, F54, E11.90		X
46	F	O	F32.8, F45.37	X	X
53	M	O	F33.1, F45.41		X
32	M	O	F40.1, F42.2		X
47	F	O	F32.1		X

*M, male; F, female; O, outpatient department; I, inpatient department; CA, desire for care vs. Autarchy; SV, conflict of self-value; SC, submission vs. Control.*

### Experimental Paradigm

#### Recruitment and Conflict Rating

Patients were approached and asked to participate in the study by their therapists either during diagnostic appointments in our outpatient unit, or during inpatient treatment. They were informed about the study and provided written informed consent in case they wished to participate. Therapists rated the occurrence of the conflict types mentioned above, and patients were scheduled for the main part of the experiment, which consisted of three phases.

(1) Association phase

Subjects were placed in front of a laptop computer (Sony Vaio, 15.5” screen). Electrodes were placed on the middle and ring fingers of the left hand for SCR measurements. On the laptop computer, 30 stimulus sentences were presented using BioTrace + V2013 software (Mind Media BV, Herten, The Netherlands). Since the software did not allow for randomized presentation of stimulus sentences, ten fixed sequences of randomly ordered stimulus sentences were created beforehand (using MATLAB’s *randperm* function), one of which was chosen at the beginning of each subject’s association phase using a 10-sided die. The same software also recorded SCR signals obtained via the recording device (Nexus-32; MindMedia BV, Herten, The Netherlands), as well as audio signals obtained via the laptop computer’s internal microphone (Realtek High Definition Audio).

Sentences were presented for 5 s each, followed by a 60 s period (indicated by a “?” on the screen) in which patients were asked to name the first three words that came to their mind, and then to associate freely until the end of the 60 s period. Participants were instructed on how to associate freely (“say anything that comes to your mind, no matter how embarrassing or senseless it may seem to you”). Audio recording started with presentation of the first stimulus sentence and continued until the end of the last trial. To facilitate assessment of reaction times (see below), a 500 Hz signal tone was played for 1 s at the onset of each sentence presentation. To minimize patients’ inhibition during free association, they were left alone in the room for the association phase of the experiment, and were assured that the experimenter would at no point listen to their recorded free associations, but only to the three words generated at the beginning of the association period.

After association to each sentence, participants were asked to complete self-paced self-ratings on 9-point Likert scales of their emotional arousal (ranging from “1: very calm” to “9: very aroused”) and their emotional valence (“-4: very negative” to “+4: very positive”), as well as their level of agreement with the sentence just presented (“1: very strong disagreement” to “9: very strong agreement”). Ratings were followed by a 30 s pause before the next sentence was presented. Before commencement of the association phase, participants were given the opportunity to get acquainted with the procedure by completing a test run consisting of 2 trials.

Stimulus sentences consisted of three categories: 6 neutral sentences, 6 generally negative sentences, and 18 conflict-related sentences. Within the conflict condition, 6 sentences were designed to express themes related to the conflict of desire for care vs. autarchy, 6 targeted conflicts of self-value, and 6 sentences tapped into the conflict of desire for submission vs. control. Most sentences were adopted from our previous studies using the free association paradigm (Kehyayan et al., 2013; Schmeing et al., 2013), only the submission-vs.-control sentences were newly generated as that conflict type had not been targeted in previous studies. Neutral sentences were designed not to elicit any specific affective responses (e.g., “I try to follow the news on a regular basis”), while the generally negative sentences were expected to provoke negative emotional reactions in most people, unrelated to personal conflict themes (e.g., “Sometimes I am frightened when I walk alone in the dark”). Conflict-related sentences were based on definitions of conflict types in OPD (OPD Task Force, 2008), which provides anchor examples of manifestations of these conflicts in different areas of life such as family, partnership, working life, and others. Examples of conflict-related sentences are: “I wish that finally someone would take care of me” for the conflict of desire for care vs. autarchy, “I often estimate myself as little competent” for conflict of self-value, and “I hate to be controlled by others” for conflict of desire for submission vs. control. See Table 2 for a complete list of stimulus sentences.

**Table 2:** Complete list of English translation of stimulus sentences.

**Neutral sentences**
Occasionally I like to watch movies on television
I try to follow the news on a regular basis
Sometimes my mood is influenced by the weather
There are topics I am more interested in than politics or economy
Mostly I do respect the traffic regulations
I find it important to find time for my hobbies once in a while
**Negative sentences**
I get annoyed when I am stuck in a traffic jam and I have an important appointment
Sometimes I am frightened when I walk alone in the dark
When an overtaking car on the other side of the road approaches me, my heart sinks into my boots
Sometimes I become sad when I think about dead soldiers in the war
Seeing a helpless animal suffer often makes me sad
When somebody is jumping the queue, it can really upset me
**Conflict sentences: desire for care vs. autarchy**
All my life I got a raw deal
I wish that finally someone would take care of me
I actually only feel good when someone is taking care of me
I give so much without really getting rewarded
I do not need anything or anybody to be happy
I hate it to be a burden for other people
**Conflict sentences: conflicts of self-value**
Usually I have a very low self-esteem
I am often embarrassed about myself
Sometimes I am disgusted by myself
I often estimate myself as little competent
I always have to struggle to be liked
I am very much dependent on other peoples’ praise
**Conflict sentences: submission vs. control**
I hate to be dominated by others
I like to tell others what to do
I do not take any orders from other people
I always play by the rules
Often I do what is demanded of me, just to have some peace and quiet
I hate to lose

(2) Break/Distraction

Following the association phase, participants had approximately 1 h to fill out several questionnaires. Questionnaires included German versions of Beck Depression Inventory II (BDI-II; Hautzinger et al., 2006), State-Trait Anxiety Inventory (STAI; Laux et al., 1981), Defense Style Questionnaire (DSQ-40; Schauenburg et al., 2007), Emotion Regulation Questionnaire (ERQ; Abler and Kessler, 2009), Childhood Trauma Questionnaire (CTQ; Wingenfeld et al., 2010), Screening for Somatoform Disorders (SOMS-2; Rief and Hiller, 2008), Toronto Alexithymia Scale (TAS-20; Bach et al., 1996), and Levels of Emotional Awareness Scale (LEAS; Subic-Wrana et al., 2001). Questionnaire data will be analyzed and presented separately.

(3) Memory recall phase

After the break/distraction, there was an unexpected memory recall. Participants were again placed in front of the laptop computer, with electrodes and sensors attached. The same 30 sentences were presented again, randomized analogously to the randomization procedure described in the association phase. This time, subjects were asked to remember and name the three words they had generated in response to each sentence. The sentences were again presented for 5 s each, followed by a “?” for 25 s, allowing a total time of 30 s per sentence to recall the previously generated associations. Trials were considered valid and included into analysis if participants had generated 3 associations at the beginning of the association phase.

#### Analysis of Reaction Times

Reaction times (RTs) were defined as the interval between onset of sentence presentation and onset of the first verbal reaction of the participant to each sentence (as in Schmeing et al., 2013; Kessler et al., 2017). For the association phase, only the onset of the first of the three generated associations was counted, resulting in one RT per trial. Reaction times were detected manually using Audacity Audio Software (Version 2.0.3^1^) by visualization of audio waveforms and identification of the interval between the 500 Hz signal tone (beginning of sentence presentation) and the first verbal response following it.

#### SCR Acquisition and Analysis

Skin conductance was measured continuously during the association phase and the memory recall phase with a sampling rate of 32 Hz. However, only SCRs recorded during the association phase were analyzed. Recordings were divided into 65 s segments representing single trials. To identify a suitable time window for SCR analysis, peak skin conductance was detected in each trial, and the mean of these peaks ± 0.5 standard deviations was chosen as time window for analysis, as in our previous analyses (Kehyayan et al., 2013; Schmeing et al., 2013). Thus, a time window from 13.3 to 33.7 s poststimulus presentation was identified and analyzed using LedaLab (Version 3.4.7; Benedek and Kaernbach, 2010). This may seem late, since SCR is often reported to peak only a few seconds after stimulus presentation (Boucsein, 1988). However, considering the protracted nature of the free association periods, it is not surprising to find a high variability in the timing of peak arousal, which may not be locked to stimulus onset. Within the response window, the maximum of phasic skin conductance activity was used as the measure of choice. Skin conductance depends on the activity of sweat glands innervated by sympathetic neurons, and thus is used as a measure of sympathetic activity (Boucsein, 1988). For illustration, we plotted SCR curves using MATLAB, with a baseline correction based on mean activity in the interval from −200 to 0 ms prior to stimulus onset. While our SCR plots suggest considerable differences in SCR between sentence conditions, for analysis, the signal was divided into tonic and phasic components of electrodermal activity, and only maximum phasic activity was compared between conditions. This explains the apparent discrepancy between SCR figures and quantitative results.

#### Statistical Analysis

First, intra-individual means were calculated for all dependent variables (memory, RT, SCR, self-rated agreement, self-rated valence, self-rated arousal) for each factor level (sentence category). To test for differences between sentence categories, we used one-way repeated measures analysis of variance (rmANOVA; with *post hoc t*-tests in case of significant results in the ANOVA). Correlations were performed intra-individually using Spearman’s rank correlation coefficient. Spearman’s R-values were then Fisher-z-transformed and tested against 0 at the group level using a one-sample *t*-test.

## Results

### General Hypotheses

**FIGURE 1:**
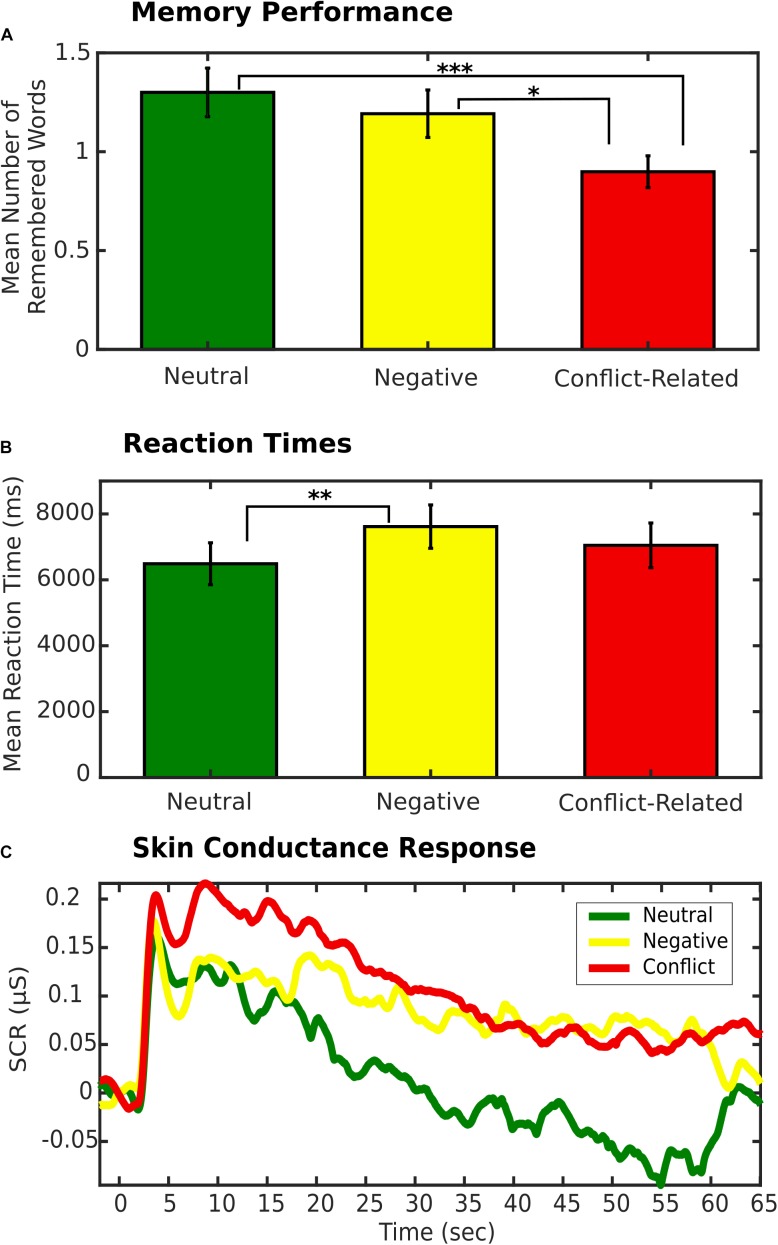
**(A)** Mean number of remembered words by sentence type. Error bars indicate s.e.m. ^∗^*p* < 0.05. ^∗∗∗^*p* < 0.001. **(B)** Mean reaction time by sentence type. Error bars indicate s.e.m. ^∗∗^*p* < 0.01. **(C)** Skin Conductance Response (SCR) by sentence type.

Motivated by our previous findings (Schmeing et al., 2013), we assessed differences in memory performance, RTs, and SCR between different sentence types (i.e., neutral, negative, or conflict-related). Clinicians’ ratings of the presumed conflict type for each patient were not taken into account in this first analysis step. Repeated measures ANOVA (rmANOVA) revealed significant differences between conditions for memory: *F*_(2, 32)_ = 5.82; *p* = 0.007 (see Figure 1A). *Post hoc t*-tests showed that associations to conflict-related sentences were forgotten more often (70% ± 2.7% [s.e.m.]) compared to negative (60% ± 4.0%; *t*_16_ = 2.50; *p* = 0.024) and neutral sentences (57% ± 4.1%; *t*_16_ = 4.02; *p* < 0.001), while memory for negative and neutral sentences did not differ (*t*_16_ = 0.75; *p* = 0.46). For RTs, too, rmANOVA showed significant differences between conditions [mean RT neutral: 6.49 s ± 0.64 s; negative: 7.62 s ± 0.66 s; conflict-related: 7.05 s ± 0.68 s; *F*_(2, 32)_ = 5.25; *p* = 0.011], with *post hoc t*-tests indicating a significant difference only between neutral and negative conditions (*t*_16_ = 3.19; *p* = 0.006), but not between conflict-related and neutral (*t*_16_ = 1.89; *p* = 0.077) or negative conditions (*t*_16_ = 1.46, *p* = 0.164), see Figure 1B. No condition effect was found for SCR [*F*_(2, 26)_ = 1.08; *p* = 0.36; see Figure 1C].

Additionally, Spearman’s rank correlations were performed intra-individually between memory and RT, as well as memory and SCR. The number of subsequently remembered words was negatively correlated with RTs [mean of Spearman’s Rs: -0.14; *t*_16_ = 2.94; *p* = 0.01 (*t*-test of Fisher-z transformed Spearman R-values tested against 0)], and SCRs (mean of Spearman’s Rs: −0.09; *t*_13_ = 2.46; *p* = 0.028).

Participants showed the highest levels of self-rated agreement with neutral sentences (6.51 ± 0.17) and lowest levels for conflict-related sentences (5.25 ± 0.26), with negative sentences in between (5.99 ± 0.32). There was a significant effect of sentence category [rmANOVA: *F*_(2, 32)_ = 7.17; *p* = 0.003], with *post hoc t*-tests showing a significant difference between neutral and conflict-related sentences (*t*_16_ = 3.98; *p* = 0.001), but not between neutral and negative (*t*_16_ = 1.90; *p* = 0.076) or negative and conflict-related sentences (*t*_16_ = 1.85; *p* = 0.083). We also observed a significant effect of sentence category on valence [*F*_(2, 32)_ = 17.96; *p* < 0.001]: Self-rated valence was highest for neutral sentences (0.53 ± 0.21), followed by conflict-related sentences (−0.50 ± 0.25), and lowest for negative sentences (−1.12 ± 0.23). *Post hoc t*-tests showed significant differences between conflict-related and neutral sentences (*t*_16_ = 4.11; *p* < 0.001), conflict-related and negative sentences (*t*_16_ = 2.34; *p* = 0.033), as well as between neutral and negative sentences (*t*_16_ = 5.23; *p* < 0.001). Finally, we observed an effect of sentence category on self-rated arousal [mean arousal neutral: 5.25 ± 0.20; negative: 6.03 ± 0.24; conflict-related: 5.57 ± 0.20; *F*_(2, 32)_ = 4.00; *p* = 0.028], with *post hoc* tests showing a significant difference only between neutral and negative sentences (*t*_16_ = 2.49; *p* = 0.024), but not between conflict-related and neutral (*t*_16_ = 1.15; *p* = 0.27) or negative sentences (*t*_16_ = 1.93; *p* = 0.071).

### Conflict-Specific Hypotheses

**FIGURE 2:**
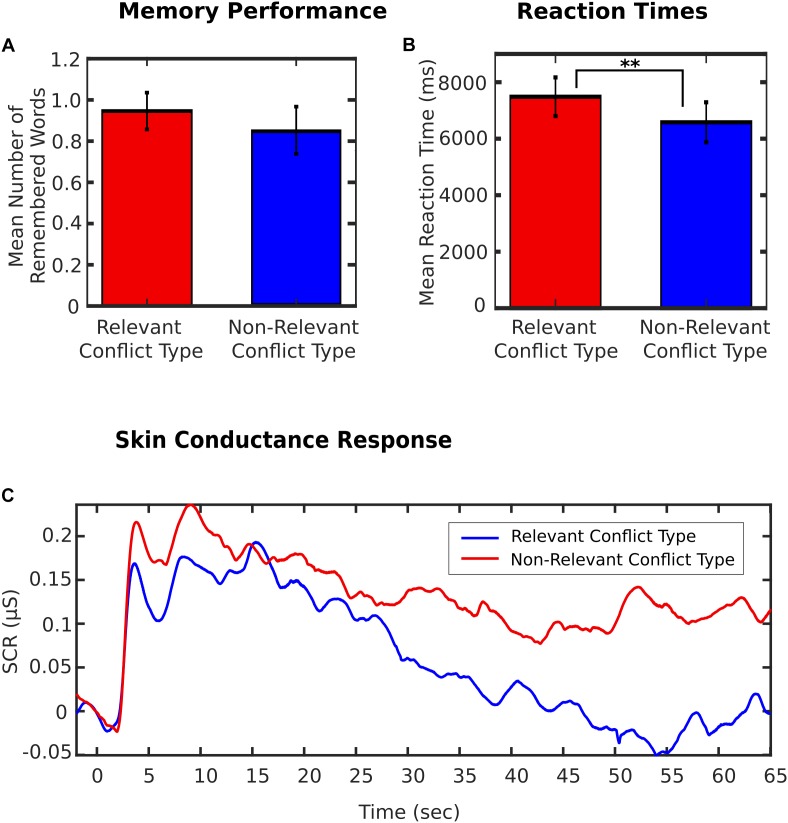
**(A)** Mean number of remembered words for personally relevant and non-relevant conflict types (based on clinical ratings). Error bars indicate s.e.m. **(B)** Reaction times (RT) for personally relevant and non-relevant conflict types (based on clinical ratings). Error bars indicate s.e.m. ^∗∗^*p* < 0.01. **(C)** Skin Conductance Response (SCR) for personally relevant and non-relevant conflict types (based on clinical ratings).

In the next step, patients’ reactions to conflict-related sentences were analyzed based on clinicians’ ratings of conflict type for each patient. Out of *N* = 17 patients, conflicts of desire for care vs. autarchy were rated to be the only relevant conflict type in 3 patients, conflicts of self-value were the only relevant conflict in 8 patients, while 6 patients showed both conflict types. No patients showed a conflict of desire for submission vs. control. For each patient, all conflict-related sentences were assigned to either of two groups: relevant conflict type(s) and non-relevant conflict type(s). Mean memory performance, RTs, and SCR were then compared across subjects between relevant conflicts and non-relevant conflicts. Reaction times were significantly longer for associations to relevant as compared to irrelevant conflicts (7.49 s ± 0.68 s vs. 6.58 s ± 0.71 s; *t*_16_ = 3.62; *p* = 0.002; see Figure 2B). No significant differences were found for memory (*t*_16_ = 0.70; *p* = 0.491; see Figure 2A) and SCR (*t*_13_ = 0.73; *p* = 0.479; see Figure 2C). There were also no differences in self-rated agreement (*t*_16_ = 0.42; *p* = 0.683), valence (*t*_16_ = 0.35; *p* = 0.732), or arousal (*t*_16_ = 1.16; *p* = 0.263) between personally relevant and non-relevant conflict types.

## Discussion

Before discussing this particular study’s results and implications, we would first like to address a fundamental epistemological question regarding the operationalization of repression and psychodynamic conflict in this study (or, more generally, of any unconscious process or psychoanalytical construct not directly accessible to observation): (How) can behavioral or psychophysiological data, such as memory or SCR, be interpreted as reflecting conflict or repression? What are the theoretical assumptions behind this claim? Aren’t there better alternative explanations of the observed behavioral and psychophysiological data? In the following, we will provide some thoughts on these questions (see also Axmacher et al., 2010; Schmeing et al., 2013, for a discussion of these issues). Historically, the idea to measure memory, reaction times, and arousal (SCR) in a free association experiment to study repression and resistance goes back to C.G. Jung’s association studies (Jung, 1906). Jung argued that stimuli that are associatively related to psychodynamic conflicts will lead to resistance that in turn is observable via autonomic arousal (i.e., increased SCR) and hesitation (prolonged RTs), and ultimately lead to re-repression of the generated material (impaired memory performance). Jung’s experimental findings were replicated repeatedly and consistently (e.g., Levinger and Clark, 1961; Köhler and Wilke, 1999). Various authors attempted to rule out alternative, more cognitive explanations (e.g., forgetting related to the number of a stimulus’ associative connections), and interpreted their results in a similar vein as Jung did. Earlier studies from our lab started out very close to these paradigms (Schmeing et al., 2013), but considered additional cognitive explanations. These included the frequency of occurrence of stimulus words and the semantic similarity between a stimulus and the given association. Follow-up studies were then adjusted to further substantiate a psychoanalytically-oriented interpretation by introducing specifically conflict-related stimulus sentences (Schmeing et al., 2013), using content analysis of audio-recorded free associations to identify signs of psychodynamic conflict (Kehyayan et al., 2013), and applied individualized stimuli generated from OPD interviews to trigger psychodynamic conflicts more reliably (Kessler et al., 2017).

Despite these efforts to clarify the validity of this experimental approach to repression, it remains inherently difficult to operationalize clinical psychoanalytic constructs such as repression, psychodynamic conflict, (counter)transference, projective identification, or conversion. This is because experiments necessarily involve some degree of abstraction as compared to the complexity of a clinical situation. While clinical experience lends some prima facie validity to our experimental design by acknowledging that arousal and hesitation (measured as SCR and RT, respectively) are indeed useful indicators of psychodynamic conflict in the therapeutic situation, these measures are obviously inherently unspecific and indirect. Therefore, interpretations based on these measures necessarily need to be considered cautiously. Nevertheless, through careful construction and stepwise advancement of experimental procedures, as described above, we believe that empirical studies can indeed operationalize relevant aspects of psychoanalytic constructs.

The present study investigated patients’ reactions in a free association paradigm designed to tap into clinically relevant psychodynamic conflicts. Earlier applications of this paradigm in healthy subjects had revealed impaired memory of free associations to conflict-related sentences, as well as behavioral (RT) and psychophysiological (SCR) signs of elevated arousal (Kehyayan et al., 2013; Schmeing et al., 2013; Kessler et al., 2017). These effects were accompanied by reduced BOLD responses in the hippocampus and increased activation of the anterior cingulate cortex in the conflict condition. The results presented here are the first in which we applied this association paradigm to a patient population.

Two types of research hypotheses were examined: First, in replication of our earlier studies, we hypothesized that patients’ behavioral (memory, RT) and physiological (SCR) reactions would show differences between neutral, negative, and conflict-related stimulus categories, regardless of patients’ individual conflict themes. Second, we proposed that there may be particularly pronounced reactions to a person’s relevant conflict type, as rated by the treating clinicians, as compared to personally irrelevant conflicts.

Concerning the first, general hypotheses, we observed an impaired memory performance for associations to conflict-related as compared to negative or neutral stimuli, as well as negative correlations of memory with both RT and SCR. However, psychophysiological reactions and RTs did not differ between trial types. Several possible factors could explain this pattern of results: Technical problems with the recording devices limited recruitment of additional patients and led to a small sample size of 14 patients for SCR analyses, making it difficult to detect possible differences, especially in signals with high variability such as SCR. Also, no difference was observed in RTs between conflict-related and other stimuli. Interestingly, in a number of trials, remarkably *short* RTs were observed when patients made negative remarks shortly after reading the sentence, like “No way!” or “Never!” This happened in 7 trials (across 5 patients), 6 of which belonged to the presumed relevant conflict type. In his 1925 essay on negation, Freud already described this kind of reaction, interpreting it as a way of acknowledging repressed contents without accepting them: “Thus the content of a repressed image or idea can make its way into consciousness, on condition that it is *negated.* Negation is a way of taking cognizance of what is repressed; indeed it is already a lifting of the repression, though not, of course, an acceptance of what is repressed” (Freud, 1925/1961). Hence, the particularly short RTs may be driven by this mechanism and do not necessarily speak against mechanisms of repression taking place while patients react to presumed conflict themes (even though this result is clearly inconsistent with our initial hypothesis). Finally, we observed a substantially larger variance of RTs across trials (i.e., within subjects) and across patients as compared to our previous studies in healthy participants (mean of RTs in current study: 7050 ms; range of standard deviations of RTs in current study: 942–5217 ms; mean of RTs in Schmeing et al., 2013: 5454 ms; range of standard deviations of RTs in Schmeing et al., 2013; 368–2239 ms; *t*_36_ = 5.09; *p* < 0.001 [individual standard deviations of RTs compared between studies using two-sample *t*-test] ) – a typical finding in clinical populations, and one which may obscure possible condition differences.

The second set of hypotheses postulated behavioral and psychophysiological reactions specifically for stimuli targeting each patient’s conflict theme(s) as rated by their treating (or interviewing) clinicians. In line with this hypothesis, we indeed observed a significant increase in RTs to putatively relevant as compared to irrelevant conflict sentences. While reaction times are a highly unspecific marker of psychodynamic processes since they depend on a multitude of various diverse cognitive processes, the observed increase in RTs is consistent with higher resistance to react to these stimuli (Jung, 1906; Levinger and Clark, 1961; Köhler and Wilke, 1999; Schmeing et al., 2013; Kessler et al., 2017). However, we did not observe any evidence for memory impairment, or signs of psychophysiological arousal for associations to conflict-specific stimuli. Apart from the technical considerations mentioned above, these findings raise questions of whether individual conflict themes were correctly identified during clinical interviews, and whether the stimulus material succeeded in triggering responses that reflect clinically relevant conflicts. Naturally, the classification of conflict themes into distinct categories is a simplification, as these themes commonly overlap in clinical reality. Furthermore, similar conflict themes may or may not have been activated (leading to observable responses) by the same triggers in different patients. For example, from two patients with a “desire for care vs. autarchy” conflict theme, one may more strongly react to a trigger like “All my life I got a raw deal” (focusing on the deficit, or the desire to be taken care of) while the other may rather react to “I do not need anything or anybody to be happy” (focusing more on the aspect of autarchy). Within the OPD system, this difference in emphasis is referred to as processing of a conflict theme in “passive mode” vs. “active mode”. While a conflict in “passive mode” is characterized by a deficit or unfulfilled desire, e.g., a desire to feel secure and be taken care of, a conflict in “active mode” is centered around defenses against this deficit or unfulfilled desire, e.g., through excessive striving for self-sufficiency and suppression of attachment wishes. In our study, individual conflict themes were classified by clinicians not otherwise involved in the experiment (and, importantly, ignorant to the stimulus material being used). Thus, there remains a possibility that the standardized stimulus sentences failed to touch upon patients’ “sore spots.” Of course, individualized stimulus sentences would have been desirable to overcome this disadvantage (as in Kessler et al., 2017; see Kessler et al., 2013, for a more in-depth discussion of the advantages of using individualized stimulus material), but this was not feasible for the current study. In this context, the observation that self-rated agreement with stimulus sentences did not differ between presumed relevant and non-relevant conflict types can be interpreted in at least three different ways: (1) the clinical rating of personally relevant conflict types was inaccurate in a substantial number of cases, (2) the stimuli used were unfit to trigger responses, although classification of relevant conflict type was correct, or (the most interesting interpretation from a psychodynamic viewpoint, but one which would need to be tested further) (3) defensive processes impaired patients’ conscious perception of their personal conflict themes, leading to impaired agreement ratings.

In our study, we did not control for previous therapeutic experiences of patients. It is reasonable to assume that as psychodynamic treatment proceeds, patients gain a better access to their specific conflict themes, possibly even leading to their eventual acceptance. This would reduce the need for regulatory processes like resistance and repression, as conflict-related contents would be less ego-threatening. In future studies, it is hence advisable to assess patients’ therapeutic record.

While failing to support our hypothesis of stronger reactions to conflict-specific cues, the current study successfully replicated earlier findings from experiments with healthy subjects showing a general tendency to react more strongly to conflict-related as compared to neutral, positive, or negative cues: Similar to the healthy participants in the studies reported in Schmeing et al. (2013) and Kessler et al. (2017), patients showed reduced memory performance for associations to conflict-related sentences. In another study in healthy subjects, we conducted a content analysis of the free associations, and compared putative markers of repression in participants with and without signs of apparent conflict in at least one trial. We found that participants with putative conflicts showed higher SCRs during and a more negative mood after association to conflict-related sentences *in general*, compared to subjects without apparent conflict (see Kehyayan et al., 2013). They also showed higher levels of self-rated agreement across all conflict-related sentences (suggesting that they were aware of these conflicts). Compared to these healthy subjects, in the current patient population, levels of agreement with conflict-related sentences were higher (5.25 ± 0.26 vs. 3.39 ± 0.20; *t*_36_ = 5.67; *p* < 0.001 [two-sample *t*-test]), and mood was more negative concerning conflict-related sentences (−0.50 ± 0.25 vs. 0.37 ± 0.19; *t*_36_ = 2.79; *p* = 0.008), i.e., the effect was more pronounced. Possibly, the tendency to repress (as indicated by reduced memory performance), or to react with psychophysiological arousal or reduced mood, is less specific to individual conflict themes than previously believed. Instead, it could be more of a personal trait or style, a way in which particular individuals react to a wider spectrum of negative cues with reference to autobiographical memories, even if they are not individually relevant psychodynamic conflicts. Further research is warranted to investigate this interpretation and its implications for clinical diagnosis and treatment.

In sum, we show that an experimental paradigm of repression based on free association can be applied in patient populations, and present some first evidence that personally relevant conflicts are processed differently.

## Author Contributions

AK, NA, HK, A-CS, and SH conceived and designed the study. AK, HK, NA, KK, and NM generated and evaluated experimental stimuli. NM and KK performed the experiments. AK, HK, NA, NM, and KK analyzed the data. AK, NA, and HK drafted the paper. A-CS, SH, NM, and KK revised the manuscript critically for important intellectual content.

## Conflict of Interest Statement

The authors declare that the research was conducted in the absence of any commercial or financial relationships that could be construed as a potential conflict of interest.

## Footnotes

1 http://audacity.sourceforge.net/
